# Triple-Negative Breast Cancer

**DOI:** 10.1155/2012/671684

**Published:** 2012-05-09

**Authors:** Quyen D. Chu, Tari King, Thelma Hurd

**Affiliations:** ^1^Health Sciences Center, Louisiana State University, Shreveport, LA 71130, USA; ^2^Department of Surgery, Memorial Sloan Kettering Cancer Center, New York, NY 10065, USA; ^3^Department of Surgery, University of Texas Health Science Centre, San Antonis, TX 78229, USA

Ever since the seminal work that was published by Perou et al., triple-negative breast cancer (TNBC) has become a common lexicon among clinicians who care for patients with breast cancer. Beside portending a poor outcome, TNBC is unique in that, unlike the hormone-positive and Her-2-positive cancer cells, it lacks target specific therapy. This is likely the result of our lack of having a clear understanding of its biology.

In this special issue, we were highly selective of only papers that we thought might further advance our understanding of the disease. Thus, the papers vary widely from the role of race/ethnicity to the metabolic/molecular influence that can potentially impact the biology of the disease. 

We hope that this special issue will serve as a focal point to continue our ongoing discussion about this challenging entity. 

*Quyen D. Chu* *Quyen D. Chu* *Tari King* *Tari King* *Thelma Hurd* *Thelma Hurd*

